# Supplementary material for: Extracellular ATP Contributes to Barrier Function and Inflammation in Atopic Dermatitis: Potential for Topical Treatment of Atopic Dermatitis by Targeting Extracellular ATP
Source: Int J Mol Sci. 2024 Nov 15;25(22):12294. doi: 10.3390/ijms252212294 (PMC11595171; doi:10.3390/ijms252212294)
Supplement: Supplementary file 1 [file ijms-25-12294-s001.zip › ijms-3311616-supplementary.pdf]

Table S1. The sequences of primer pairs are shown.

human primer for	forward	reverse
FLG	CATGGCAGCTATGGTAGTGCAGA	ACCAAACGCACTTGCTTTACAGA
β-actin	ATTGCCGACAGGATGCAGA	GAGTACTTGCGCTCAGGAGGA
mouse primer for	forward	reverse
Tarc	AGGTCACTTCAGATGCTGCTC	ACTCTCGGCCTACATTGGTG
Ifn-γ	CCATCAGCAACAACATAAGCGTCA	CCGAATCAGCAGCGACTCCTT
Il-4	GATCCGGATGGTCCCATTCTA	CTCTGCAGCTCCATGAGAACACTA
Il-22	CCATACATCGTCAACCGCACCT	GAAGGCAGGAAGGAGCAGTTCTT
Il-33	TGCAGGAAAGTACAGCATTCAAG	TTGGTCTTCTGTTGGGATCTTCTTA
Tslp	GGCGACAGCATGGTTCTTC	GCTCGAACTTAGCCCCTTTCA
Tbp	ACTCCTATGACCCCTATCACTCCTG	GTTTACAGCCAAGATTCACGGTA
mouse primer for	probe	
Il-13	Mm00434204_m1	
